# Age-Related Changes in the Functional Network Underlying Specific and General Autobiographical Memory Retrieval: A Pivotal Role for the Anterior Cingulate Cortex

**DOI:** 10.1371/journal.pone.0082385

**Published:** 2013-12-18

**Authors:** Pénélope Martinelli, Marco Sperduti, Anne-Dominique Devauchelle, Sandrine Kalenzaga, Thierry Gallarda, Stéphanie Lion, Marion Delhommeau, Adèle Anssens, Isabelle Amado, Jean François Meder, Marie-Odile Krebs, Catherine Oppenheim, Pascale Piolino

**Affiliations:** 1 Laboratory of Memory and Cognition, Université Paris Descartes, Boulogne-Billancourt, France; 2 Center of Psychiatry and Neurosciences, INSERM UMR S894, Université Paris Descartes, Paris, France; 3 Department of Radiology, Université Paris Descartes, Centre de Psychiatrie et Neuroscience, INSERM U894, Paris, France; 4 Laboratory of Physiopathology of Psychiatric Diseases, Centre Hospitalier Sainte Anne, Paris, France; 5 Institut Universitaire de France; University Of Cambridge, United Kingdom

## Abstract

Age-related changes in autobiographical memory (AM) recall are characterized by a decline in episodic details, while semantic aspects are spared. This deleterious effect is supposed to be mediated by an inefficient recruitment of executive processes during AM retrieval. To date, contrasting evidence has been reported on the neural underpinning of this decline, and none of the previous studies has directly compared the episodic and semantic aspects of AM in elderly. We asked 20 young and 17 older participants to recall specific and general autobiographical events (i.e., episodic and semantic AM) elicited by personalized cues while recording their brain activity by means of fMRI. At the behavioral level, we confirmed that the richness of episodic AM retrieval is specifically impoverished in aging and that this decline is related to the reduction of executive functions. At the neural level, in both age groups, we showed the recruitment of a large network during episodic AM retrieval encompassing prefrontal, cortical midline and posterior regions, and medial temporal structures, including the hippocampus. This network was very similar, but less extended, during semantic AM retrieval. Nevertheless, a greater activity was evidenced in the dorsal anterior cingulate cortex (dACC) during episodic, compared to semantic AM retrieval in young participants, and a reversed pattern in the elderly. Moreover, activity in dACC during episodic AM retrieval was correlated with inhibition and richness of memories in both groups. Our findings shed light on the direct link between episodic AM retrieval, executive control, and their decline in aging, proposing a possible neuronal signature. They also suggest that increased activity in dACC during semantic AM retrieval in the elderly could be seen as a compensatory mechanism underpinning successful AM performance observed in aging. These results are discussed in the framework of recently proposed models of neural reorganization in aging.

## Introduction

The study of autobiographical memory (AM) constitutes an ecological approach to investigate long term declarative memory based on real-life events. AM contains information that is specific to an individual. It encompasses both episodic autobiographical memories (EAM), which consists of specific events in a particular spatio-temporal context, for which we can mentally travel back in time, and re-experience the encoding context, and semantic autobiographical memory (SAM), which stores general knowledge of the self as well as general events, information we are aware of in the absence of specific recollection [1]–[4]. According to constructive models of AM, episodic details are generally accessed through generative controlled processes [1], [5], [6] involving executive functions such as selection of relevant and inhibition of irrelevant information [7]–[8], and the maintenance and manipulation of this information in working memory [9]–[11] to handle a coherent AM retrieval.

Converging lines of evidence suggest that the healthy elderly are generally less efficient in retrieving EAM, while their capacity to retrieve SAM is well-preserved [12]–[16]. More precisely, the healthy elderly have a reduced capacity of recollection of specific events, regularly providing general events. Even when they do retrieve a specific event, they often fail to recollect spatiotemporal and phenomenological details. A dysfunction of generative processes seems responsible for the reduction in the recollection of EAM with age [12], [15], [17], [18]. Accordingly, AM performance has been found to largely depend on an age-related decline in working memory [17], [19]. More recently, we reported that the elderly showed increasing difficulty to access deeper levels of specificity of AM and that this effect was mediated by performance on executive functions (i.e. updating and inhibition) and working memory [20]. These findings are in keeping with the well established age-related deficits in prefrontal functions [21]–[22].

Neuroimaging studies of EAM retrieval in young adults have repeatedly reported activation of a fronto-temporo-parietal network extending into the medial prefrontal cortex (MPFC), the posterior cingulate cortex (PCC), limbic structures, the angular gyrus and the precuneus [23]–[25]. Few neuroimaging studies have investigated the specific neural substrates of EAM compared to SAM [26]–[28]. While reporting common activation among the two kinds of memory retrievals, these studies reported that EAM recruited to a greater extent medial temporal lobe structures as well as different prefrontal regions encompassing the dorsolateral (dLPFC), the ventrolateral (vLPFC), the dorsomedial (dMPFC) and the ventromedial (vMPFC) prefrontal cortex, the anterior cingulate cortex (ACC), as well as posterior cortical structures. Interestingly, these prefrontal regions and the anterior cingulate cortex are known to play a pivotal role in cognitive control and are associated with executive functions [29]–[31]. The extent of activation of the vLPFC and the ACC in the initial access phase during EAM retrieval has been shown to be related to the specificity of memories and control processes such as inhibition in young adults [32]. Thus, these results add evidence to the fact that EAM strategic retrieval is implemented in frontal regions that are, in turn, engaged in executive processes [6]. They also suggest that a decline of the cognitive processes implemented in these prefrontal regions could be related to the AM retrieval deficits observed in older adults.

Little is known about the neural underpinning of AM decline in aging. Neuroimaging studies converge in showing that the network subserving EAM is quite resilient in the elderly, but subtle differences have also been reported [33]–[37]. Some studies indicated greater activity for elderly subjects in the right hippocampus [35], the fusiform gyrus and occipital areas [34] during EAM retrieval, while other studies showed reduced recruitment of medial temporal regions and the precuneus [33], or the vLPFC [36]. Moreover, St. Jacques et al. [36] reported a reduced top-down modulation from the vLPFC, a region subserving strategic processes, to the hippocampus during EAM retrieval. Finally, St. Laurent et al. [37] reported a similar pattern of activation between young and old participants, but older subjects showed a less specific neural signature of EAM compared to other memory tasks (i.e., standard episodic and semantic retrieval).

These contradictory results could be explained by methodological differences. For example, studies reporting hyper-activations in the elderly recorded autobiographical memories prior to the scanning session to create personalized AM retrieval cues [34]–[35], that could have resulted in a re-encoding of the memory trace. Moreover, Maguire and Frith [35] used a recognition task that could have minimized the engagement of strategic retrieval processes. On the other hand, studies reporting hypo-activations in the elderly used general cues (words or pictures) for the AM task [33], [36], [37], requiring maximal cognitive control load. Thus, age differences in these studies could depend on the degree of engagement of executive functions that, in turn, could influence the activation of the AM network. Moreover, to date, a direct comparison between SAM and EAM retrieval processes in the elderly has not been considered since all aforementioned studies compared an autobiographical and a non-autobiographical memory task.

The aim of the present study was to fill this gap by requiring healthy young and elderly subjects to retrieve EAM and SAM while recording their brain activity by means of fMRI. In accordance with the theoretical hierarchical models of AM retrieval that stress the increasing role of executive functions to access specific memories (e.g., [6]), with the cognitive mechanisms of EAM deficits in aging (e.g., [20]), as well as with previous neuroimaging AM studies in aging (e.g., [37]), we made several hypotheses. At the behavioral level, we predicted that EAM retrieval should be more related to executive functions in young and older adults than SAM retrieval. We expected spared SAM and impoverished EAM specificity in older adults. This effect should be related to a decline of executive functions. At the neural level, during EAM retrieval, older subjects should show lower activation in frontal regions, compared to young adults. In turn, activity in these regions should be linked with the decline of executive functions and the specificity of memories. During SAM retrieval, since it represents the prevalent access level of AM [6], thus engaging less strategic retrieval, and is spared in the elderly, we expected to find comparable activations among the two groups.

## Methods

### Participants

All participants gave their informed written consent and the study received agreement of the local ethic committee of Sainte Anne Hospital (CPP Ile de France 3 n°2687). Twenty healthy young (YA) (25–45 years old, mean =  29.2±5.5,10 women) and 17 healthy old (OA) adults (65–80 years old, mean = 68.2±4.4,10 women) all right-handed (according to the Edinburgh Handedness Inventory; [38]), and native French speakers participated to the study. Medical, demographic, and psychometric data were obtained prior to the scanning session. All participants were unmedicated, living at home and rigorously screened for uncontrolled hypertension and cerebrovascular risk factors. Exclusion criteria included presence of history of alcohol or substance abuse, head trauma, major disease affecting brain function, neuropsychiatric disorders (tested with the Mini-International Neuropsychiatric Interview, [39]), depression (tested with Geriatric Depression Scale, [40], cut-off score >10; YA: 2.65±2.53 and OA: 3.52±2.60), abnormal general cognitive functioning as assessed by the Mattis scale ([41], cut-off score <136; YA: 142.47±1.17 and OA: 140.17±2.98), and abnormal visual mental imagery ability (short form of Minnesota Paper form Board [42]; cut-off score < 2; YA: 4.40±0.80 and OA: 4.11±0.78). Moreover, they all performed on their normal age range for episodic memory as assessed by the Grober and Buschke's test [43] (z scores of sum of 3 total recalls, delayed total recall; YA: 0.02±0.18, 0.01±0.01 and OA: 0.61±0.99, 0.24±0.65). The number of years of education was 16.55±2.21 for YA and 13.41±3.55 for OA, the two groups were matched according to their verbal abilities and crystallized intelligence as assessed by the Mill Hill test [44] (a multiple-choice synonym vocabulary test; percentile score: 56,64±17,23 and 59.41±24.61, t(35) =  –.27, p = .78).

### Neuropsychological Measures

In order to characterize the executive and working memory functions, we used the recommendation of Miyake et al. [45], who proposed the distinction of three elementary executive processes: “shifting”, “updating”, and “inhibition” of inappropriate responses. Consistently, we administered to the participants the following standard tests (see [20]), for details): the trail making test ([46], TMT B-A); running span ([47]; total score), and the Stroop test ([48]; interference score), to assess shifting updating and inhibition functions respectively. Other measures of the basic working memory functions were obtained using the classic digit and visuospatial spans [49] and a multimodal span [50]. For correlation purpose, all scores were transformed into z scores and scaled in the same direction, so that higher scores reflect better performance.

### Materials and Procedure

#### General Organization

The experiment comprised two major phases. During the first phase (pre-scanning interview), participants were tested for exclusion and inclusion criteria, they underwent medical examination, neuropsychological assessment and completed the Taste and Interest Questionnaire (TIQ) that was employed to create personal cues used in the scanning session. In the second phase (scanning session), participants were trained in the experimental task outside the scanner, then made the task in the scanner and finally re-evoked their memories during the debriefing.

#### Pre-scanning interview

In the pre-scanning interview, exclusion and inclusion criteria were verified by means of a clinical exam and psychometric tests, and then neuropsychological tests and the TIQ were submitted to subjects. The aim of the TIQ was to collect information enabling to create personalized cue for each participant without directly asking for descriptions of past memories to avoid the re-encoding of memories [33], [51]. Participants were informed that the purpose of the questionnaire was to obtain a description of their personality thanks to information about their main life interests up to five years ago. They had no prior knowledge of the aim of the fMRI task, preventing the possibility for participants of searching for memories linked to their taste and interests between the two sessions. The questionnaire concerned their personal lives and consisted of a list of 220 interests (e.g.: leisure, food, drink, transport, places where they lived, holidays, jobs, studies). For each item, the participants had to answer whether it was personally pertinent or not, rated by 1 and 0 respectively. When an item was pertinent, they had to rate how important (from 0 to 10) and frequent (Frequent/Rare) the activity or interest had been in their life.

#### Personal items elaboration

Twenty-four activities or interests for EAM and SAM were selected from the TIQ based on the pertinence (the score should be 1) and the personal importance (scored ≥5). These activities or interests were used to create the cues for the scanning session. Cues for each condition were made up according to the frequency of the activity. For EAM, we selected those activities that were scored as rare, while for SAM we selected activities scored as frequent. For EAM, the sentences used as cues had the following structure: “a unique memory linked to…” followed by the selected events for this category. For SAM, the sentences used as cues had the following structure: “a habit of the past linked to…” followed by the selected events for this category.

#### Experimental task

The participants were first invited to take part to a training session before the fMRI scanning. Participants received detailed explanations on the nature of the task and participated at a brief simulation of the experiment on a laptop. Three tasks were explained to the subjects: EAM and SAM retrieval and a control task called scene imagery task (SI).

- EAM was defined as a memory of a single event that occurred at a specific time and place, of short duration, lasting less than 24 hours. Participants were instructed to mentally relive personal episodes prompted by cues and to try to retrieve spatiotemporal, affective and perceptual details (such as time, location, perceptions, feelings, scenery, and people present in the scene) (e.g.: ‘‘a unique memory linked to a trip in New York”).

- SAM was defined as a memory of a repeated event that occurred several times in the past, a regular activity that used to occur at a routine time and place or a memory of an extended event that may describe a summary of events over several days, weeks, or months without a precise moment in time (e.g.: ‘‘an habit of the past about your dancing lessons’’).

- SI control condition consisted in a sentence completion task (e.g.: “the postman delivers …”). The subjects were instructed to first complete the sentence with the first word that comes in mind (e.g.: " the mail") and then imagine the scene described in the sentence in a peculiar context, the North Pole. This scenario was used due to the non-personal character of the North Pole since we wanted to avoid any references to SAM and EAM in this condition. Moreover, participants were explicitly instructed to imagine scenes with no reference to their personal life including events or past habits. Contrarily to others experiments using either general semantic or perception tasks (e.g., sentence completion and size discrimination; word definition; image generation of an object), we wanted to isolate processes specific to AM retrieval thanks to the control condition that necessitated general semantic and scene construction (as AM) but with no reference to personal information (e.g.,[51]).

For the two AM retrieval conditions, participants were asked to press a button as soon as they gained access to a memory. For the SI, participants were told to press the button as soon as they completed the sentence and begun to visualize the imagined scene in the context of the North Pole. After instructions, participants were trained on three trials for each condition (EAM, SAM, SI) with the experimenter providing feedback concerning the pertinence of each response. The cues used for the trainings were different from those used during the scanning session.

#### Scanning Session

During scanning, cues were visually presented in white font on a black background projected on a screen viewed by means of a mirror incorporated into the head-coil. E-Prime software (Psychology Software Tools, Inc., Pittsburgh) in combination with an Integrated Functional Imaging System (IFIS) was used for the presentation and timing of stimuli and collection of response. Responses were made on an MR-compatible two-buttons box.

Participants completed four functional scans, each lasting 9 min 16 seconds, in a single session. Each functional scan was composed of 18 items (6 EAM, 6 SAM and 6 SI) presented in a randomized order within mini-blocks of 3 items of the same condition. Each trial lasted 27 s with the following time-course: the cue was presented for 5 s, followed by a white cross at the center of the screen for 19 s, then the cross turned red for 3 s informing the participants of the end of the present trial and the arrival of the next one.

#### Post scan interview

Participants were asked to recall again each EAM and SAM previously retrieved in the scanner in order to check that memories met minimal criteria for the corresponding category (single events, situated in time and place, lasting less than 24 hours; e.g., "I remembered my visit in the Coliseum in Rome with my wife and my daughter one afternoon of July, ten years ago ", or repeated or extended events; e.g., "I recalled my walks in the woods with my dog Cachou"). The subsequent analyses were performed only on memories that met all the above mentioned criteria. We computed the proportion of EAM and SAM (i.e., memories respecting minimal criteria and for which the participants had pressed a button indicating retrieval) on the total number of trials seen under the scanner.

Moreover, EAMs were rated for richness and specificity on standard scales [3], [12]–[13]. More precisely, the presence of a sense of remembering with recall of specific spatial and temporal details, and other contextual and phenomenological details in each evocation was noted (1 point by type of detail, max. 4; e.g., " I remembered my visit in the Palace of Tokyo in Paris, in August 2009, as if I was still there, being together with Chiara in a room of the exhibition in the first floor in the dark to see the TV reports and talking with other visitors, it was 6 pm and still very warm, but it was worth it!, after then we settled down in the restaurant of the outdoor museum in front of the Seine..."). We computed for each participant a global ratio of specificity (EPI score) totaling up the sum of spatiotemporal, other contextual and phenomenological details divided by the sum depending on the number of EAM.

## fMRI Method

### 

#### MRI data acquisition

All data were acquired with a 3 T scanner (Discovery MR 750, General Electric Healthcare). The anatomical scan used an inversion recovery 3-D T1-weighted gradient-echo sequence images (TE = 4.3 ms, TR = 11.2 ms, TI = 400 ms, matrix = 384×384, slice thickness = 1.2 mm). Functional images were acquired using a gradient echo echoplanar (EPI) sequence (TE = 30 ms, TR = 2000 ms, flip angle = 90°, matrix = 64×64, slice thickness = 3 mm, 42 contiguous sections). The first four volumes of each functional run were discarded in order to allow longitudinal magnetization to approach equilibrium.

#### Pre-processing of fMRI data

All data were processed using SPM5 software (Statistical Parametric Mapping 5, Welcome Dept. Cognitive Neurology, UK; www.fil.ion.ucl.ac.uk/spm). Standard pre-processing procedures were applied to MRI data. EPI volumes were corrected f or slice timing, realigned to the first image, co-registered with the high-resolution T_1_-weighted image and normalized into a standard stereotaxic space. The normalization used the Montreal Neurological Institute (MNI) template and the rigid transformations computed during the segmentation of the high-resolution T_1_-weighted image. Finally, the normalized EPI volumes were smoothed using an isotropic Gaussian kernel filter of 5 mm full-width half-maximum.

#### Statistical first level analysis of fMRI data

Only responses meeting criteria for the EAM and SAM conditions were used for the subsequent analyses. A trial was considered as a hit if (1) the participant had pressed the button during the trial (indicating retrieval) and (2) the description of the memory during the debriefing corresponded to the targeted type of memory (see above). For the control condition, a trial was considered as a hit if the participant had pressed the button during the trial and the description of the scene imagery during the debriefing confirmed non-self reference.

Memory retrieval (i.e. access or strategic research phase) was modeled by convolving the time period between cue presentation and the subjects’ response with the hemodynamic response function (HRF). For each subject, General Linear Model was used to estimate the parameters of interest. Parameters of movement were also included in the model as regressors of no interest. Whole brain t-tests were computed to estimate the contrasts of interest for each subject: EAM vs. SI and SAM vs. SI. Then, contrasts for each individual were used for second-level analyses.

#### Statistical second level analysis of fMRI data

We first computed whole brain t-tests to evidence areas significantly activated in the different conditions separately for the two groups. The following comparisons were tested: EAM vs. SI and SAM vs. SI. Contrast for these analyses were carried out using cluster level correction with the false discovery rate (FDR) with p<0.05 (voxel-wise statistical threshold was fixed at p<0.001 uncorrected). We used an inclusive conjunction between the activation maps resulting from the comparisons between the conditions of interests, EAM and SAM, and SI in the two groups to mask subsequent between group analyses (threshold for each individual contrast were set at p(FDR)<0.05 at the cluster level, voxel-wise threshold at p<0.001 uncorrected). The rationale of this choice was that we were interested in differences between conditions and groups in areas showing an actual activity in a least one condition and group and not in spurious differences that could result from areas showing deactivation. Then, we conducted a 2×2 ANOVA with group (YA, OA) and condition (EAM, SAM) as factors. Statistical threshold for this analysis was set at p<0.001 not corrected for multiple comparison with an extended threshold of k = 20. The same ANOVA was computed on an *a priori* region of interest (ROI) on the hippocampus, a region that is known to be involved in episodic autobiographical retrieval (e.g., [52]–[59]). This ROI was defined using the template of the Anatomical Automatic Labeling (AAL), [60]. For this *a priori* ROI we used a more lenient threshold of p<0.01 uncorrected.

#### Correlation

We extracted percentage signal change for the cluster showing a significant group by condition interaction (see results section), using Marsbar toolbox, and computed for each group correlations between signal change in this region and the neuropsychological and autobiographical scores (i.e., EPI, EAM, SAM) outside SPM using STATISTICA8©. For correlation purpose, all scores were transformed into z scores and scaled in the same direction, so that higher scores reflect better performance.

## Results

### Behavioral Results

The results of neuropsychological measures and autobiographical scores are reported in **Table 1**. They showed a global difference between YA and OA in all executive and working memory scores in favor of YA.

**Table 1:** Neuropsychological and autobiographical measures according to the group.

	YA	OA	t-value (df = 35)
**Neuropsychological scores**			
INHIB	6.57 (±0.46)	17.79 (±0.88)	–6.36***
TMTB-A	28 (±15.39)	58.29 (±26.96)	–4.39***
R-SPAN	14.25 (±3.12)	11.06 (±3.61)	2.88**
WM	52.05 (±3.08)	37.11 (±3.08)	5.61***
**Autobiographical scores**			
% SAM	91.11 (±7.77)	87.01 (±8.50)	1.36
RT-SAM	2.24 (±1.06)	2.96 (±1.61)	–1.63
% EAM	90.42 (±7.77)	89.22 (±8.47)	0.45
RT-EAM	2.28 (±0.94)	2.80 (±1.71)	–1.15
EPI score	81.06 (±8.73)	73.41 (±12.88)	2.14*

* p<.05; ** p <.01; *** p <.001 INHIB (interference score Stroop, inhibition). TMTB-A (trail making test B-A score, shifting). R-SPAN (running span, updating). WM ( digit, visuospatial and multimodal spans, working memory). EAM and SAM (percentage of correct responses); RT (response time); EPI (Episodic score of EAM).

Both young and old participants showed a high percentage of correct trials and a rapid response time. The percentage of correct trials and response time for EAM and SAM conditions did not differ between the groups, but YA had a higher EPI score than OA.

The correlations are reported in **Table 2**. EPI score correlated positively with inhibition, updating and working memory performance in YA, while it was positively correlated with inhibition and shifting in OA, even if in OA only a marginally significant correlation between EPI and shifting survived after correction for multiple comparisons using FDR. In both groups, there was no correlation with the percentage of correct trials (EAM, SAM), nor the response time (data not shown).

**Table 2:** Correlations between EPI score percentage of correct AM responses and neuropsychological scores in both groups.

	EPI	EAM	SAM	INHIB	TMT B-A	R-SPAN	WM
**EPI**	-	0.088	0.446	**0.532**	**0.647**	0.109	0.478
		p = .745	p = .083	**p = .034**	**p = .007^$^**	p = .687	p = .061
**EAM**	0.158	-	**0.698**	–0.171	0.146	–0.284	–0.004
	p = .517		p = .003*	p = .525	p = .588	p = .269	p = .988
**SAM**	0.113	**0.693**	-	–0.137	0.114	–0.451	0.149
	p = .646	p = .001**		p = .613	p = .675	p = .069	p = .582
**INHIB**	**0.623**	–0.045	–0.198	-	**0.539**	0.349	**0.578**
	p = .004*	p = .853	p = .415		**p = .025**	p = .184	**p = .019**
**TMT B-A**	0.438	0.205	0.343	0.286	-	0.096	0.258
	p = .061	p = .399	p = .150	p = .234		p = .723	p = .335
**R-SPAN**	**0.516**	0.239	0.110	**0.495**	0.429	-	0.314
	**p = .024^$^**	p = .323	p = .653	**p = .031^$^**	p = .059		p = .238
**WM**	**0.659**	0.010	0.035	**0.575**	**0.486**	**0.769**	-
	p = .002*	p = .716	p = .703	p = .010*	**p = .035^$^**	p = .0001**	

*p<0.05, **p<0.01, ^$^p<0.06. EPI (Episodic score of EAM); EAM and SAM (percentage of correct responses); INHIB (interference score Stroop, inhibition). TMTB-A (trail making test B-A score, shifting). R-SPAN (running span, updating). WM (digit, visuospatial and multimodal spans, working memory). The correlations of the top are the ones of old group, those of the bottom are the ones of young group. Asterisks denote correlations that survived FDR correction,

### fMRI Results

#### Within Group Analyses

**Figure 1:**
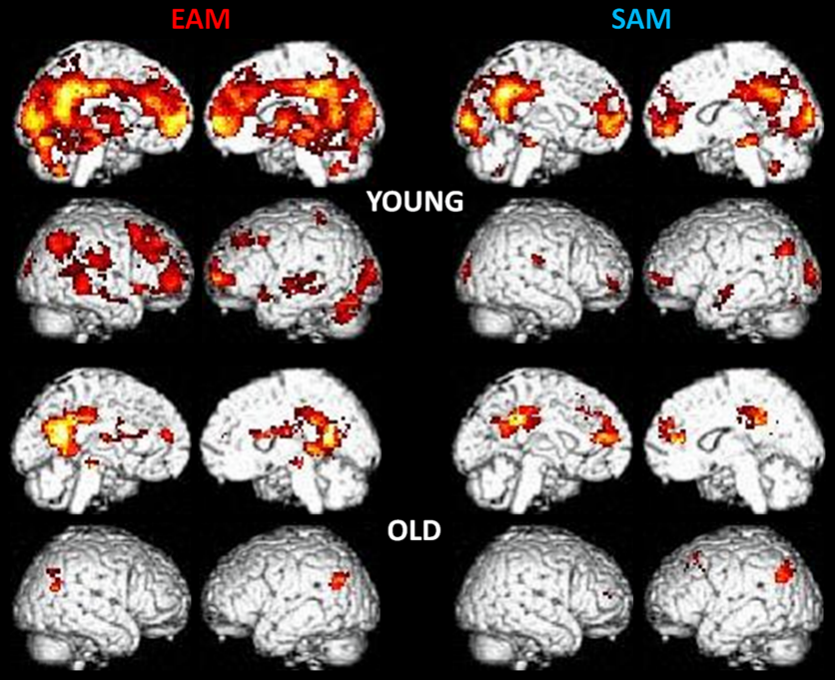
Main results for the EAM and SAM conditions in young and old groups. Statistical maps are superimposed to an MNI T1 template. Statistical threshold was set at p<0.001 (uncorrected) with an extended threshold of k = 20.

For the EAM condition in the YA we reported activations in a large cluster encompassing lateral and medial frontal regions, and posterior medial regions. In particular, we found activation in cortical midline structures encompassing the medial prefrontal cortex (MPFC), the anterior cingulate cortex (ACC), the middle cingulate cortex (MCC), the posterior cingulate cortex (PCC), and precuneus. Moreover, the inferior parietal and occipital regions as well as medial temporal regions comprising the hippocampus were significantly activated. For the SAM condition a similar pattern of activation was evident but less extended (**Figure 1**).

For the OA a less extended set of regions were activated. For the EAM condition we mainly reported activity in cortical midline structures such as the MPCF, the ACC, the middle cingulate cortex (MCC), and the precuneus. We also found activation in occipital and middle temporal regions, in the caudate nucleus, and in the thalamus. A less extended pattern of activity was reported in the SAM condition, with additional activation in the superior frontal gyrus (**Figure 1**).

The list of local activation maxima for each condition in both groups using cluster level correction with the false discovery rate (FDR) with p<0.05 is reported in **Table 3** **.**

**Table 3:** Contrast for each group and both conditions using cluster level correction with the false discovery rate (FDR) with p<0.05.

						MNI		
	Side	Labels	BA	k	t	x	y	z
YOUNGEAM								
	L	MCC	23	10923	11.17	–9	–39	36
	L	Pre-Cun	23		10.74	–9	–54	30
	R	MPFC	10		10.69	6	57	3
	L	Ang. Gyr.	39	291	10.39	–51	–69	27
	L	Ang. Gyr.	19		6.68	–42	–72	39
	L	Mid. Temp.	39		5.53	–57	–60	21
	R	Ang. Gyr.	7	387	6.80	36	–66	42
	R	Inf. Par.	40		5.90	42	–51	39
	R	Ang. Gyr.	39		5.85	54	–60	30
YOUNGSAM								
	L	Pre-Cun	23	2018	8.86	–6	–54	33
	R	Cun.	17		7.96	12	–93	9
	L	MCC	23		7.81	–9	–39	36
	L	Mid. Front.	10	791	6.97	–9	48	–6
	R	Mid. Front.	10		6.31	9	42	–3
	R	vMPFC	11		5.91	18	48	–9
	R	Cereb.	30	208	6.91	18	–36	–15
	L	pHipp.			5.98	–15	–27	–15
		Cereb.			5.48	0	–24	–21
	R	Rolandic	48	44	6.06	45	–24	24
	R	Cereb.		54	5.75	9	–54	–42
	L	Ang. Gyr.	39	98	5.11	–45	–69	33
	L	Ang. Gyr.	39		3.68	–48	–54	27
	L	Mid. Temp.	48	44	4.65	–54	–12	–6
	L	Mid. Temp.	22		4.61	–57	–6	–12
	L	Mid. Temp.	21		4.29	–54	0	–21
OLDEAM								
	R	Lingual	27	1430	7.38	9	–45	–3
	L	Pre–Cun	27		7.36	–12	–39	3
	L	Calcarine	29		6.93	–12	–48	9
	L	Thalamus		131	6.13	–18	–9	12
	L	Caudate			5.19	–18	3	30
	L	Caudate			4.87	–15	27	18
	R	Ang. Gyr	39	54	6.11	48	–66	39
	R	Mid. Temp.	39		5.72	51	–60	21
	R	Ang. Gyr	39		4.19	45	–54	27
	L	Hipp.	30	60	5.39	–18	–18	–12
	R	Lingual	27		5.20	9	–27	–9
	L	Ang. Gyr	39	86	4.98	–48	–60	30
	L	Mid. Temp.	39		4.36	–48	–60	21
	L	MPFC	32	44	4.83	–9	51	18
OLDSAM								
	L	Ang. Gyr	39	90	5.65	–48	–63	39
	L	Mid. Occ.	7		3.71	–36	–75	39
	L	Sup. Front.	9	397	5.43	–21	27	39
	L	ACC	32		4.83	–9	51	12
	R	ACC	32		4.67	9	36	12
	L	MCC	23	370	5.27	–12	–36	30
		MCC			5.05	0	–30	42
		MCC			4.99	0	–36	36

=  Left; R  =  Right; BA  =  Brodmann Areas; ACC  =  anterior cingulate cortex; Pre-Cun  =  precuneus; sup. MPFC  =  superior medial prefrontal cortex; vMPFC  =  ventral medial prefrontal cortex; Ang. Gyr  =  angular gyrus; Hipp.  =  hippocampus; Cereb.  =  cerebellum; Inf. Par.  =  inferior parietal lobule; Mid. Temp.  =  middle temporal gyrus; MCC  =  middle cingulate cortex; Mid. Occ.  =  middle occipital gyrus; Mid. Front.  =  middle frontal gyrus; pHipp  =  parahippocampus; Mid. Front.  =  middle frontal gyrus; Sup. Front.  =  superior frontal gyrus; Inf. Par.  =  inferior parietal lobule. L

#### Between Group Analyses

To test for differences between groups we carried out a mixed 2×2 ANOVA with group (YA/OA) as between subject factor and condition (EAM and SEM) as within subject factor.

**Figure 2:**
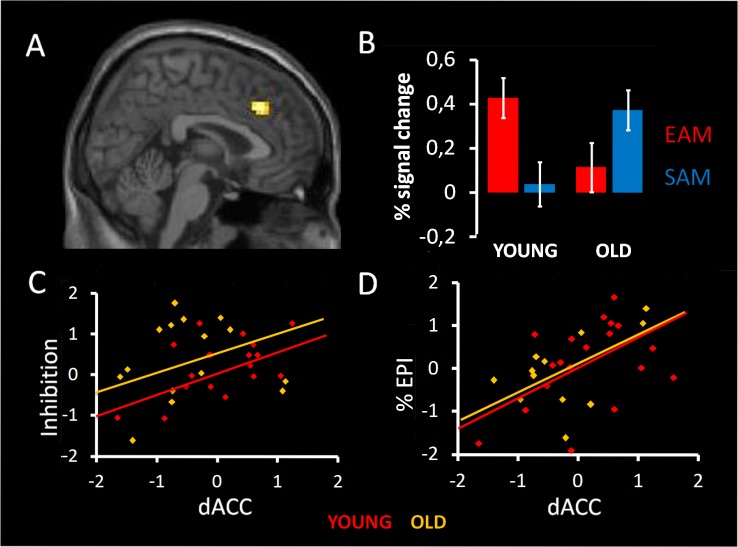
Results of the 2×2 ANOVA with condition (EAM/SAM) and group (YA/OA) as factors. A) Main effect of group by condition in the dorsal anterior cingulate cortex (dACC); B) Plots represent percentage of signal change for each condition of interest and both groups;. C) Correlations between dACC activated in EAM condition and inhibition score in the two groups; D) Correlations between dACC activated in EAM condition and EPI score (richness of specificity) in the two groups. Statistical maps are superimposed to an MNI T1 template. Statistical threshold was set at p<0.001 (uncorrected) with an extended threshold of k = 20.

The whole brain analysis did not reveal neither a main effect of group, nor a main effect of condition (p<0.001 not corrected for multiple comparison with an extended threshold of k = 20). Nevertheless, we found a significant interaction between the two factors in the dACC (BA32; coordinates of local maxima: x = 3; y = 27; z = 36, p<0.001 not corrected for multiple comparison with an extended threshold of k = 20). Post-hoc LSD comparison on signal change showed that activity in dACC was greater in the EAM compared to SAM condition in YA (p = 0.001) and that the opposite pattern was evident in OA (p = 0.015). Moreover, activity in dACC was greater in YA compared to OA (p = 0.03) in EAM condition, and inversely, in the SAM condition activity was greater in OA compared to YA (p = 0.021), see **Figure 2** **A, B**.

**Figure 3:**
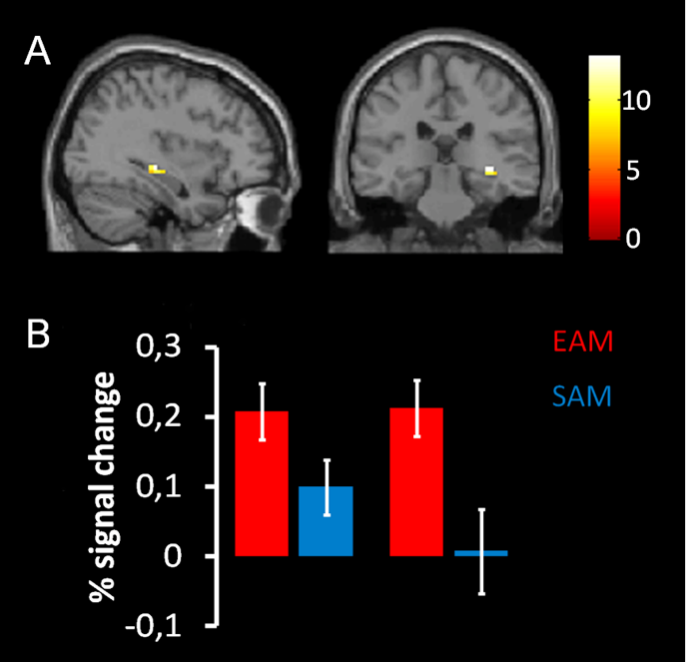
Results of the 2×2 ANOVA with condition (EAM/SAM) and group (YA/OA) as factors in the right hippocampus using region-of-interest analyses. A) Main effect of condition showed in the sagittal and the coronal plane on the left and the right respectively. B) Plots represent percentage of signal change in right hippocampus (36 –27 –6) for each condition of interest, red bars represent EAM, blue bars represent SAM, and both groups, young and old group on the left and right respectively. Statistical maps are superimposed to an MNI T1 template.

The same analysis on the ROI in the hippocampus, revealed a significant main effect of the condition (p = 0.004, coordinates of local maxima: x = 36; y = –27; z = –6). This effect was due to the fact that this structure was more activated in the EAM compared to the SAM condition independently of the group (**Figure 3** **A, B**).

#### Correlation

We reported a correlation between the percent signal change in the dACC (that presented a group by condition interaction), and autobiographical and neuropsychological scores, for each group and each condition (see **Table 4** for detailed results).

**Table 4:** Correlations between the dACC activations and the EPI score percentage of correct AM responses and neuropsychological scores in both groups.

	EPI	EAM	SAM	INHIB	TMT B-A	R-SPAN	WM
**dACC-EPI YA**	**0.602 p = .006**	–0.072 p = .770	–0.350 p = .141	**0.491 p = .033**	0.232 p = .340	0.208 p = .392	0.210 p = .389
**dACC-EPI OA**	**0.531 p = .034**	–0.089 p = .743	0.190 p = .479	**0.547 p = .028**	0.242 p = .365	0.009 p = .973	**.547 p = .028**
**dACC-SAM YA**	0.031 p = .899	–0.205 p = .398	–0.404 p = .086	–0.080 p = .744	–0.131 p = .591	–0.124 p = .611	–0.273 p = .258
**dACC-SAM OA**	0.402p = .122	–0.098p = .716	0.191p = .478	0.308p = .244	0.201p = .455	0.081p = .765	0.413p = .112

EPI (Episodic score of EAM). EAM and SAM (percentage of correct responses); INHIB (interference score Stroop, inhibition). TMTB-A (trail making test B-A score, shifting). R-SPAN (running span, updating). WM (digit, visuospatial and multimodal spans, working memory). None of the correlations survived FDR correction.

Regarding the EAM condition, a positive correlation was reported between EPI score and the dACC, which, in turn, correlated with inhibition performance in both age groups (**Figure 2** **C, D**). Another positive correlation was reported in OA between working memory and the dACC. There was no correlation concerning the SAM condition in both groups. Nevertheless, none of this correlation survived after correction for multiple comparisons using FDR (**Table 4**).

## Discussion

The aim of this study was to shed light on the effect of aging on the neural network supporting AM retrieval. In particular, for the first time, we sought to explore, in aging, the relationship between episodic and semantic components of AM, executive functions, and neural activity by the means of fMRI. At the behavioral level, we confirmed that EAM retrieval is impoverished in aging and that this decline is related to the reduction of executive functions [19]–[20]. At the neural level, EAM retrieval in aging showed lower activation in a frontal region underpinning cognitive control, the dACC, which, in turn, correlated with executive functions and the specificity of memories regardless of the age of the participants. Most interestingly, we evidenced a significant interaction in the dACC that showed a greater activation during EAM, compared to SAM, in young adults and a reversed pattern in the elderly. In the following, we will discuss the implications of our results and will try to integrate them into existing neurocognitive models of aging.

As expected, we reported activation in a well-known network implicated in AM retrieval encompassing medial temporal structures (hippocampus and parahippocampus), anterior and posterior cortical midline structures and lateral prefrontal regions [23]–[25], [61]. In the elderly, this network was more restrained and mainly comprised midline cortical regions. Hence, when subtracting a non-self scene construction task (control task), which requires intentional access to long term declarative memory, and visual imagery like AM retrieval, the main activations concern a core network engaged in self-referential processing [24], [62]. This finding highlights the connection between AM and the self-representation [1], [2], [6] and argues in favor of its relative maintenance in the elderly [13], [63].

When directly comparing the activation for specific and general autobiographical events’ retrieval in the two groups, we confirmed that EAM is specifically dependent on the hippocampus [23]–[28], and we extended this finding to the elderly [35], [58]–[59]. Interestingly, focusing on the access phase, like in the present study, Ford et al. [26] showed that activity in regions such as the lateral prefrontal cortex and the medial temporal lobe (including the dLPFC and the hippocampus) was uniquely engaged by specific events relative to general events. These results are in line with the Multiple Trace Theory (MTT, [54]) which argues in favor of a permanent involvement of the hippocampus in the retrieval of remote episodic autobiographical memories, while semanticized memories’ retrieval would not be specifically dependent on this structure. Our findings suggest that age-related effects on the neural reorganization do not alter this distinctive involvement of the hippocampus in specific versus general AM retrieval.

Concerning previous neuroimaging findings on AM retrieval in the elderly, our results confirmed that the core network underpinning this function is relatively maintained in aging, even if it appears restrained to some pivotal regions. Nevertheless, when directly comparing the two groups, subtle differences appeared. The main difference between EAM and SAM was evidenced in the dACC showing a dissociative pattern of activation in the young and the elderly: greater activation in EAM in the former and greater activation in SAM in the latter. In other words, the dACC showed lower activation in EAM retrieval in the elderly. In turn, the level of activity in the dACC correlated with executive functions and specificity of memories in both groups (but, none of the correlation survived FDR correction). These findings seem in contrast with AM studies reporting hyper-activation [34]–[35], but in accordance with those demonstrating hypo-activation [33], [36], in the elderly. These divergences could be explained by methodological differences between our and previous studies as already discussed in the introduction. Studies reporting hyper-activation seem to use a EAM task that could have resulted in a lower demanding task (e.g., recognition versus recollection of specific details). Interestingly, here we reported hypo-activation during the access phase of EAM, extending the findings of St Jacques et al. [36] that identified hypo-activation of the ventral LPFC during EAM retrieval during the late elaboration phase. In keeping with our correlation analyses, they also reported that this age-related hypo-activation was attenuated for memories for which the elderly succeeded to reach high episodicity, and activity in this region correlated with executive functions (verbal fluency performance). Hence, our results should not be seen in contrast, but as complementary with previous reports, unraveling the role of the dACC in the EAM strategic research phase, executive functions, and their decline in aging.

Although AM retrieval has been frequently associated with cortical midline activations [51], [64], the exact role of the ACC - a region in the medial wall of the frontal lobe which is known to be fundamental to the integration of cognitive and affective processes - in accessing AM has never been a subject of specific debate. As previously mentioned, neuroimaging studies on EAM have pointed to the increasing activity of the frontal lobe associated with increasingly specific AMs, but mostly concerning lateral regions [26]–[27]. More specifically, the dLPFC is assumed to play a pivotal role in the controlled access phase of specific AM [26], [65]–[66]. Several researches have reported that emotion and cognition are processed in a ventral and a dorsal sub-region of the ACC respectively, the latter corresponding to the region found in our study [29], [67]. The dorsal component is involved in various higher-order cognitive processes including different executive functions [68]–[71], and cognitively demanding tasks requiring stimulus–response selection in the face of competing streams of information [29], [67], [72]. This role of the dACC is coherent with its specific implication in EAM retrieval and with its link with the richness of specific details (episodic score) found here, since these memories are built up *via* generative processes which include strategic, evaluative and inhibitive processes [1], [6], [65]. The correlation between the dACC activity and the episodic and the inhibition scores of young and older participants could be in line with its frequently reported activation during the Stroop task [29], [67] and suggests that probably inhibitory processes, more than other executive functions, are strongly recruited during the initial search of a specific memorie. Moreover, it is also consistent with studies that report age-related reduction of metabolism in the dACC [73]. Thus altogether, the pattern of activation of the dACC in EAM strategic retrieval suggests a central role of frontal/executive processes in the difficulty of reaching a higher level of AM specificity in aging [20]. Nevertheless, further evidence should be provided to confirm the present findings, since most of the correlations did not remain significant when correction for multiple comparisons was applied.

Most interestingly, we also reported that, contrarily to young adults, the elderly showed higher activations of the dACC in the SAM condition relative to the EAM condition. This activation was not correlated with executive functions, thus it is difficult to argue that the SAM condition needed more strategic resources for older adults than for young adults. How do we explain this finding? In the last years, several models of neural reorganization in aging have been proposed. These models differ fundamentally in their predictions of hypo- or hyper-activation in the elderly and in their link with behavioral performances. We would like to note here that none of these models has been developed to specifically explain AM performance, so we did not expect to find a direct match between our results and the predictions of a specific model. Our results, indeed, seem to partially meet the prediction of several models. For example, some of them [74]–[75] predict compensatory mechanisms in the form of an over-recruitment of different brain regions reflecting a diminished neural efficiency to attain preserved performance in specific domains. Even if we did not report any general over-activation in the elderly, the greater activity of the dACC during SAM, a memory task that is largely known to be spared in the elderly, could reflect this sort of compensation. In the same vein, our results seem coherent with the Compensation-Related Utilization of Neural Circuit (CRUNCH) hypothesis [76]–[79]. This model, developed in the working memory framework, states that the elderly would engage more neural resources (over-recruitment) than young adults when facing a task requiring a low level of cognitive load (reaching soon their resource limitation), and they would have fewer resources available to meet the processing requirements of more demanding tasks (which would lead to a decline of performance and hypo-activations). Data consistent with the CRUNCH model has been reported in the prefrontal cortex during working memory tasks that differed in the number of items that had to be kept in mind [76]. Here, while we did not report general differences between the two groups, greater dACC activity in elders is observed during SAM, that could be seen as a less demanding cognitive task following AM retrieval models [1], [6], [20]. Indeed, fewer strategic retrieval processes are needed to reach this level of retrieval. Coherently, we did not find any correlation between SAM retrieval and executive functions in both groups. By contrast, the diminished engagement of the dACC during EAM in elders could follow the overrun of cognitive resources due to the complexity of the task. Therefore, the prediction of the CRUNCH model could account for the differential activation of the dACC according to the AM retrieval task at hand.

In summary, we showed that the AM retrieval network is strongly resilient in aging. Nevertheless, some subtle differences emerged when comparing the nature of the retrieved material in term of the specific/generic nature of AM. In addition to the well-known role of frontal regions in strategic research processes, our findings highlight that the dACC seems to play a pivotal role in EAM retrieval in young adults due to its central role in cognitive control and especially in inhibition. These findings give further support to the link between executive functions and AM retrieval, and specify that the dACC could be a central node for these processes. The diminished activity of the dACC could explain the difficulty of the elderly to recollect specific details. Following AM models, indeed, if cognitive resources are limited, the hierarchical memory search could prematurely be interrupted at a more general level of specificity. On the other hand, increased activity in the dACC during SAM could be seen as a compensatory mechanism underpinning successful SAM performance observed in aging. The present findings provide new directions for future work that aim to determine the role of executive control and anterior cingulate activation in overgeneral AM recall beyond normal aging.
